# Development and evaluation of a model to predict herd-level milk fat concentration in Irish spring-calving grazing dairy herds

**DOI:** 10.3168/jdsc.2025-0976

**Published:** 2026-05-15

**Authors:** C. Heffernan, E. Ruelle, M. Dineen

**Affiliations:** Teagasc, Animal and Grassland Research and Innovation Centre, Moorepark, Fermoy, Co. Cork, Ireland, P61 P302

## Abstract

The objective of this quantitative study was to mathematically reparametrize a herd-level milk fat concentration model, based on grazing rotation, into a continuous day-of-year model. An additional objective was to evaluate the predictive performance of the reparameterized model using milk recording data from 5,165 spring-calving (Feb. to Apr.) herds in Ireland. The dataset included 13,788 individual herd-level milk sample recordings collected between February and November 2023. The grazing-rotation coefficients from the original model were aligned to their corresponding day-of-year, and continuous daily coefficients were generated using linear extrapolation. The daily coefficients were then integrated into the original multivariable linear regression model, replacing grazing rotation with day-of-year. The reparameterized model was initially evaluated for prediction accuracy and precision using the evaluation dataset reported by Heffernan et al. (2025a), and it demonstrated strong predictive ability (R^2^ = 0.78; root mean square error [RMSE] = 0.24%; concordance correlation coefficient [CCC] = 0.85). Model prediction accuracy and precision were further assessed using a national dataset of 5,165 spring-calving herds with intermittent milk recording records from February to November 2023. The model also demonstrated strong predictive ability for the spring-calving dataset (R^2^ = 0.65; RMSE = 0.32%; CCC = 0.76). These results demonstrate that the reparameterized day-of-year model accurately predicts herd-level milk fat concentration across the grazing season (Feb. to Nov.) for Irish spring-calving dairy herds. The model provides a practical tool for distinguishing expected seasonal variation in milk fat concentration from other mechanisms that reduce milk fat synthesis, thereby supporting informed management decisions and enhancing production efficiency.

## Highlights

• Continuous day-of-year model developed to predict herd-level milk fat concentration across the grazing season.
• Demonstrated strong predictive accuracy using both the development dataset and a national dataset of 5,165 spring-calving herds.
• Provides a practical tool for differentiating expected seasonal milk fat patterns from other mechanisms that reduce milk fat synthesis in pasture-based systems.

## Graphical Abstract

This study developed a continuous day-of-year model to predict herd-level milk fat concentration throughout the grazing season in Irish spring-calving dairy herds. A previously published grazing-rotation model was mathematically reparametrized by aligning rotation coefficients with their corresponding day-of-year and generating continuous daily coefficients through linear extrapolation. These were incorporated into the original multivariable linear regression framework, allowing seamless prediction using only sampling date and herd milk fat concentration predicted transmitting abilities. The model demonstrated strong predictive performance when tested on the original dataset (coefficient of determination [R2] = 0.78; root mean square error [RMSE] = 0.24%; concordance correlation coefficient [CCC] = 0.85) and maintained robust accuracy when applied nationally to 5,165 spring-calving herds (R2 = 0.65; RMSE = 0.32%; CCC = 0.76). By distinguishing expected seasonal patterns from other mechanisms that reduce milk fat synthesis, the model provides a practical decision-support tool for managing milk composition and identifying herds experiencing impaired milk fat production. ICBF = Irish Cattle Breeding Federation.

**Figure:**
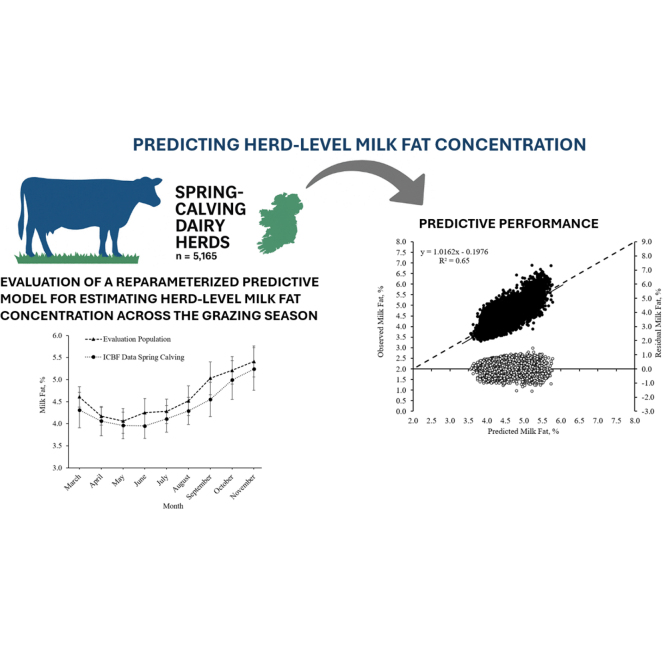

Milk fat is one of the most variable components of milk (McCarthy et al., 2018), and due to its importance in determining milk's economic value (Mohan et al., 2021), its variability is a major concern for dairy farmers. In Ireland, the milk fat concentration of spring-calving dairy cows varies considerably throughout the grazing season (Lahart et al., 2024). A consistent decline is observed from early spring (Feb.–Mar.) to early summer (May–Jun.), with an absolute reduction of 0.39% recorded during 2025 (CSO, 2026). Recognizing these patterns at the herd level can be challenging due to herd dynamics (e.g., cows entering and exiting the herd over time due to calving and culling) and genetic gain. Therefore, identifying whether a herd is achieving its expected genetic potential not only ensures production efficiency but also enables farmers to respond effectively to instances of diet-induced reductions in milk fat synthesis.

Quantifying diet-induced reductions in milk fat synthesis, or milk fat depression (**MFD**), has been approached in several different ways (Carty et al., 2017). In an Irish context, Neville et al. (2023) defined MFD as a bulk tank milk fat concentration of <3.30% accompanied by a corresponding milk protein concentration >3.20%. However, this definition does not account for herd genetic potential. Herds with high milk fat PTA might be unlikely to fall below these thresholds, yet they might still experience substantial reductions in milk fat concentration across the grazing season. Developing a tool capable of predicting herd-level milk fat concentration throughout the grazing season, while accounting for genetic potential, is therefore particularly important for spring-calving dairy herds, as it would help identify periods of diet-induced reductions in milk fat synthesis.

Previously, Heffernan et al. (2025a) developed a multivariable linear regression model to predict bulk tank milk fat concentration using variables including grazing rotation and the herd's PTA for milk fat concentration. However, a limitation of the original model was its reliance on discrete grazing rotations, which can be ambiguous across grazing seasons and vary between farms with factors such as pasture growth rates and regional conditions. Therefore, the objectives of this quantitative study were (1) to mathematically reparameterize the original model by Heffernan et al. (2025a) into a continuous day-of-year model capable of predicting herd-level milk fat concentration throughout the grazing season and (2) to evaluate the predictive performance of the reparameterized model at a national scale using milk recording data from 5,165 spring-calving herds.

A detailed description of the original model development is outlined in Heffernan et al. (2025a). Briefly, an observational experiment was conducted over a 2-yr period, involving 25 commercial spring-calving dairy herds. Each herd was visited 10 times per year, corresponding to 10 individual grazing rotations. During each visit, feed and bulk tank milk samples were collected, as well as information on farm management practices and herd genetic characteristics. The data from 12 of the 25 herds were used for model development. Univariate analyses were performed to examine associations between individual explanatory variables and milk fat concentration. Variables with *P* < 0.2 were subsequently included in a multivariable linear regression model, which was refined using backward stepwise elimination until only variables with *P* < 0.05 remained. The final multivariable linear regression model consisted of grazing rotation and the herd's milk fat concentration PTA. The predictive accuracy and precision of the model were evaluated using data from the remaining 13 herds across all 10 grazing rotations. The model predicted bulk tank milk fat concentration with an R^2^ of 0.79 and a root mean square error (**RMSE**) of 0.23%. The slope of the relationship between observed and predicted bulk tank milk fat concentrations was 1.09 (SE = 0.04), with an average bias of 0.05%.

In the current study, the grazing-rotation coefficients developed by Heffernan et al. (2025a) were mathematically transformed by aligning them with the average day-of-year on which each grazing rotation occurred during the commercial farm study (mean ± SD: rotation 1 = 72 ± 4 d; rotation 2 = 107 ± 4 d; rotation 3 = 128 ± 4 d; rotation 4 = 152 ± 1 d; rotation 5 = 170 ± 3 d; rotation 6 = 191 ± 3 d; rotation 7 = 215 ± 7 d; rotation 8 = 236 ± 7 d; rotation 9 = 275 ± 10 d; rotation 10 = 313 ± 7 d). Continuous daily coefficients were generated using linear extrapolation between the successive grazing rotations, producing a continuous, piecewise linear function. The daily coefficients were then integrated into the original multivariable linear regression model, replacing grazing rotation with day-of-year and enabling continuous prediction of milk fat concentration across the grazing season. The reparameterized model was evaluated for prediction accuracy and precision initially using the Heffernan et al. (2025a) evaluation dataset and subsequently using a national dataset of 5,165 spring-calving herds, which was obtained from the Irish Cattle Breeding Federation. The dataset contained spring-calving herds that calved from January to April 2023, with intermittent milk recording records that were collected from February to November 2023. On average, each herd was sampled 2.7 times across the sampling period. Only data from herds with a minimum of 35 cows recorded per sampling time point and within 3 SD of the mean were retained. The final dataset consisted of 5,165 dairy herds and 13,788 individual herd-level milk sample recordings. Prediction accuracy and precision were evaluated using regression analysis (PROC REG in SAS version 9.4, SAS Institute Inc., Cary, NC). Evaluation criteria included the coefficient of determination (R^2^), average bias, slope between observed and predicted milk fat concentrations, mean square error, RMSE, relative prediction error, and concordance correlation coefficient (**CCC**), as described by Lin (1989), Rook et al. (1990), Fuentes-Pila et al. (1996), and Zom et al. (2012).

The descriptive statistics of the herd characteristics, milk recording timing, and milk composition of the national spring-calving dataset are presented in Table 1. The number of cows sampled per herd averaged (mean ± SD) 108 ± 70. Although this is less than the average herd size reported by Heffernan et al. (2025a; 234 ± 101), it is consistent with the average herd size reported in the National Farm Survey (NFS, 2023; 97 cows). In the current spring-calving dataset, the herd average milk fat concentration PTA was 0.11% ± 0.06%, which is lower than that reported by Heffernan et al. (2025a; 0.19% ± 0.04%). The average milk fat concentration in the spring-calving dataset was 4.33% ± 0.54%, with a range of 3.28% to 6.86%. The average was again lower than that observed by the Heffernan et al. (2025a; 4.50% ± 0.52%) evaluation dataset; however, the range reported by the Heffernan et al. (2025a) evaluation dataset was narrower (3.14% to 5.97%). The monthly average milk fat concentrations across the grazing season for Heffernan et al. (2025a) and the current spring-calving dataset are presented in Figure 1A. A curvilinear trend was observed across the grazing season and was consistent among both datasets. This aligns with previous experiments investigating pasture-based systems (O'Callaghan et al., 2016; Lahart et al., 2024).

**Table 1:** Descriptive statistics of herd characteristics, milk recording timing, and milk composition in spring-calving herds (n = 5,165) calving between January and April 2023 (inclusive), with milk recording records from February to November 2023 (inclusive)

Item	n	Mean	SD	Minimum	Maximum
Number of samplings per herd	5,165	2.7	2.0	1.0	36.0
Number of cows sampled per herd	13,788	108	70	35	631
Herd average calving date	13,788	18/2	—	10/1	15/4
Milk record sampling date	13,788	25/6	—	04/2	30/11
Herd average PTA					
Milk fat, %	13,779	0.11	0.06	−0.10	0.47
Milk protein, %	13,779	0.09	0.03	−0.06	0.22
Herd average milk composition					
Milk fat, %	13,560	4.33	0.54	3.28	6.86
Milk protein, %	13,681	3.59	0.33	2.69	4.86

**Figure 1:**
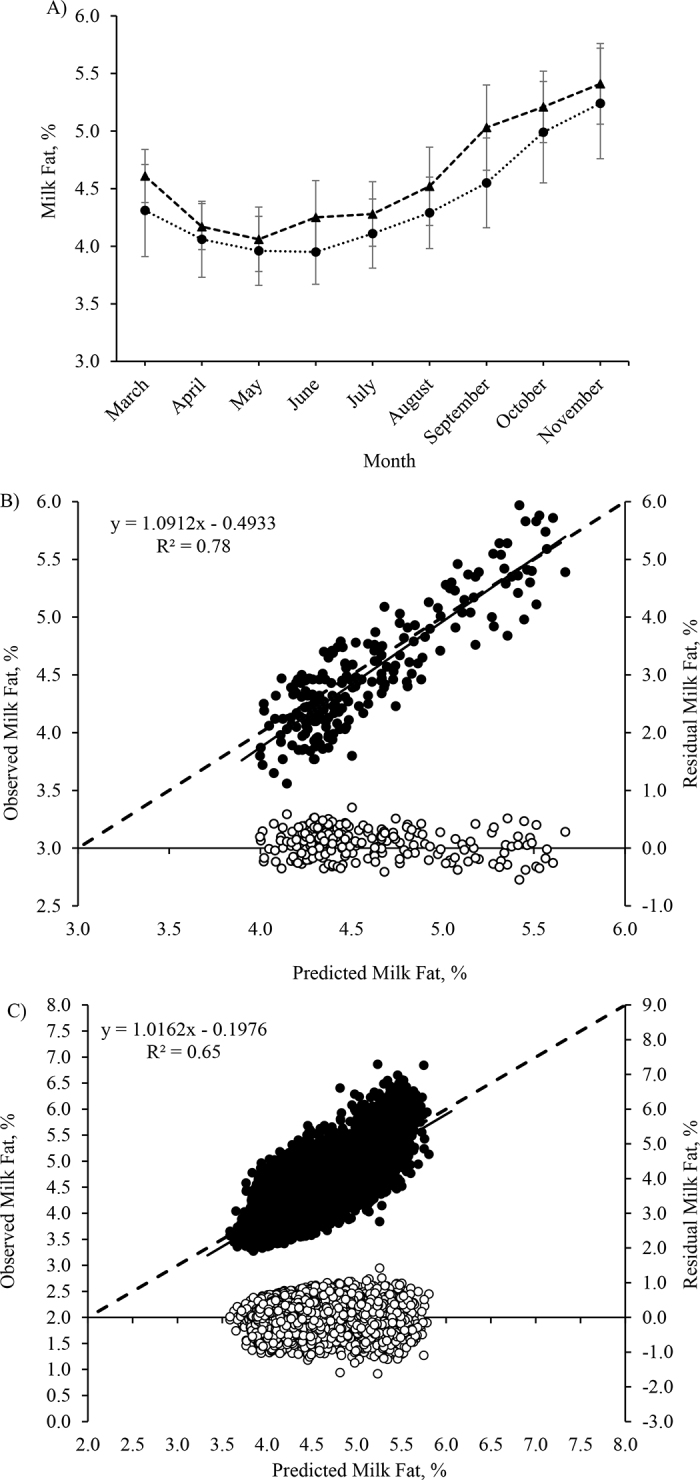
(A) Herd-level milk fat concentration (mean ± SD; %) across the grazing season for the Heffernan et al. (2025a) dataset (▲) and the spring-calving dataset comprising 5,165 herds (●); (B) Heffernan et al. (2025a) observed milk fat (%) versus herd-level milk fat (%) predicted by the reparameterized multivariable linear regression model (●); and (C) spring-calving dataset observed herd average milk fat (%) versus herd-level milk fat (%) predicted by the reparameterized multivariable linear regression model (●). In both (B) and (C), the solid line (—) represents the linear regression, and the dashed line (- - -) is the unity line. Conditional residuals are also presented (○).

Statistics describing the prediction accuracy and precision of the reparameterized model for milk fat concentration, compared with observations from the Heffernan et al. (2025a) dataset and the current spring-calving dataset, are presented in Table 2, Figure 1B and 1C. For the Heffernan et al. (2025a) evaluation dataset, the reparameterized model predicted a mean milk fat concentration of 4.59% ± 0.41%, with values ranging from 3.99% to 5.67%. Regression analysis presented in Figure 1B demonstrated R^2^ of 0.78 and RMSE of 0.24%, with an average bias of 0.07 (Table 2), indicating strong predictive ability of the reparameterized model. A similar R^2^ of 0.79 was reported by Heffernan et al. (2025a), suggesting comparable explanatory power between models. Although the model residuals were normally distributed, the slope between observed and predicted milk fat concentrations was 1.09 (SE = 0.04), suggesting a slight tendency to overestimate lower milk fat concentrations. Nevertheless, the strong Pearson correlation (r = 0.88) and high CCC (0.85) demonstrate robust overall predictive performance. For the current spring-calving dataset, the reparameterized model predicted a mean milk fat concentration of 4.46% ± 0.43%, with values ranging from 3.59% to 5.81%. The regression analysis presented in Figure 1C demonstrated a R^2^ of 0.65 and RMSE of 0.32%. The reparameterized model also had a bias of 0.13 and a slope close to unity of 1.02, suggesting that the predictions were closely aligned with the observed values. The reparameterized model maintained a strong Pearson correlation (r = 0.80) and high CCC (0.76), indicating robust overall predictive performance.

**Table 2:** Prediction accuracy and precision of the model for milk fat concentration compared with observations from the Heffernan et al. (2025a) dataset and the current spring-calving dataset^1^

Dataset	Mean observed	Mean predicted	Bias	b	R^2^	MSE	RMSE	RPE (%)	Pearson correlation	CCC
Heffernan et al. (2025a) dataset^2^	4.51	4.59	0.07	1.09	0.78	0.06	0.24	5.33	0.88	0.85
Current spring-calving dataset^3^	4.33	4.46	0.13	1.02	0.65	0.10	0.32	7.41	0.80	0.76

1 b = slope of the regression of observed and predicted milk fat concentration; MSE = mean square error; RMSE = root mean square error; RPE = relative prediction error; CCC = concordance correlation coefficient.

2 Evaluation dataset comprising data from 13 spring-calving dairy herds across 10 grazing rotations over a 2-yr period (Heffernan et al., 2025a).

3 Current spring-calving dataset comprised data from spring-calving herds (n = 5,165) calving between January and April 2023 (inclusive) with milk recording records from February to November 2023 (inclusive).

Expanding the prediction model to use continuous day-of-year coefficients rather than discrete grazing-rotation categories enhances its applicability across the grazing season and reduces ambiguity associated with defining rotation boundaries. In addition, the variables of sampling date and herd milk fat concentration PTA are readily accessible to farmers, supporting the potential for wide-scale adoption. Given that over 95% of dairy farms in the National Farm Survey operate spring-calving, pasture-based systems (O'Brien et al., 2018), the model has broad national relevance. Moreover, 80% of Ireland's annual milk production occurs between March and September (NMA, 2024), demonstrating the importance for managing milk composition during the grazing season. Although milk fat concentration follows a consistent seasonal pattern (CSO, 2026), episodes of substantially reduced milk fat synthesis (i.e., >0.4%) continue to pose challenges for farmers (Neville et al., 2023). The updated predictive model provides a valuable decision-support tool that enables producers to estimate the expected herd milk fat concentration, based on genetic potential, for a given date and to identify deviations that could indicate impaired milk fat synthesis, requiring further investigation. As interventions to correct reductions in milk fat concentration can be costly (Heffernan et al., 2025b,c), distinguishing normal seasonal variation from genuine nutritional or metabolic issues is essential for maintaining profitability.

Heffernan et al. (2025a) reported a milk fat concentration range of 3.56% to 6.09%, meaning that some of the observed values in the current spring-calving dataset fall outside this predictive range, which might partly explain some of the reduced model prediction accuracy and precision when compared with the Heffernan et al. (2025a) evaluation dataset (Table 2). Similarly, the milk fat concentration PTA range reported by Heffernan et al. (2025a; 0.08% to 0.26%) was narrower than the ranges observed in the current spring-calving dataset (−0.10% to 0.47%), which could further account for the slightly lower prediction accuracy and precision of the reparameterized model for the spring-calving dataset. This emphasizes the need for caution when applying the model beyond the boundaries of the development dataset. The model is intended for use in spring-calving grazing dairy herds, as both the model development and evaluation datasets consisted exclusively of spring-calving dairy herds. Stage of lactation is known to influence milk fat concentration independent of nutritional and seasonal effects (Walker et al., 2004); therefore, further investigation is needed to assess prediction accuracy and precision in split-calving or autumn-calving herds. The bias observed between the reparameterized model predictions and the current spring-calving dataset observations also warrants further investigation. In particular, the reparameterized model was developed using bulk tank milk measurements, whereas the current spring-calving dataset is based on milk recording data, which can differ when samples are composited to the herd-level. Furthermore, the current spring-calving dataset is limited to a single year of data. Given that production outcomes can vary between years due to differences in environmental conditions and pasture nutritive value, additional years of data would further strengthen the analysis. Altogether, the model explained a substantial proportion of the variation in milk fat concentration (R^2^ = 0.65); however, some variability remained unexplained (RMSE = 0.32%). For a typical Irish spring-calving system producing ~5,500 kg of milk per cow annually, this level of unexplained variation could correspond to ~17 to 18 kg of milk fat per cow per year. This highlights the economic importance of improving our understanding of the remaining sources of variation in milk fat concentration in pasture-based dairy systems.

Nonetheless, the results from this study demonstrate that the reparameterized day-of-year model accurately predicts herd-level milk fat concentration across the grazing season for Irish spring-calving dairy herds. By distinguishing expected seasonal variation in milk fat concentration, based on genetic potential, from other mechanisms that reduce milk fat synthesis, the model offers a practical tool to support informed management decisions and improve production efficiency.
